# Treatment of a Textile Effluent from Dyeing with Cochineal Extracts Using *Trametes versicolor* Fungus

**DOI:** 10.1100/tsw.2011.99

**Published:** 2011-05-05

**Authors:** Gabriela Arroyo-Figueroa, Graciela M. L. Ruiz-Aguilar, Leticia López-Martínez, Guillermo González-Sánchez, Germán Cuevas-Rodríguez, Refugio Rodríguez-Vázquez

**Affiliations:** ^1^ Departamento de Ingeniería Agroindustrial, División de Ciencias de la Salud e Ingenierías, Campus Celaya-Salvatierra, Universidad de Guanajuato, Salvatierra, Gto., Mexico; ^2^ Departamento de Química, División de Ciencias Naturales y Exactas, Campus Guanajuato, Universidad de Guanajuato, Guanajuato, Gto., Mexico; ^3^ Centro de Investigación en Materiales Avanzados, Chihuahua, Chih., Mexico; ^4^ Departamento de Ingeniería Civil, División de Ingenierías, Universidad de Guanajuato, Guanajuato, Gto., Mexico; ^5^ Departamento de Biotecnología y Bioingeniería, Centro de Investigación y de Estudios Avanzados, IPN, México, D.F., Mexico

**Keywords:** fungus, degradation, anthraquinone, effluent

## Abstract

*Trametes versicolor* (Tv) fungus can degrade synthetic dyes that contain azo groups, anthraquinone, triphenylmethane polymers, and heterocyclic groups. However, no references have been found related to the degradation of natural dyes, such as the carminic acid that is contained in the cochineal extract. Experiments to determine the decolorization of the effluent used in the cotton dyeing process with cochineal extract by means of Tv fungus were done. Treatments to determine decolorization in the presence or absence of Kirk's medium, glucose, and fungus, with an addition of 50% (v v_-1_) of nonsterilized effluent were performed. Physicochemical characterization was performed at the start and end of the treatment. Degradation kinetics were determined. A direct relationship was found between the dry weight of fungi, pH, and the decolorization system, with higher decolorization at lower pH levels (pH ~4.3). High decolorization (81% ± 0.09; 88% ± 0.17; and 99% ± 0.04) for three of the eight treatments (Kirk's medium without glucose, Kirk's medium with glucose, and without medium with glucose, respectively) was found. Toxicity tests determined an increase in the initial effluent toxicity (7.33 TU) compared with the final treatment (47.73 TU) in a period of 11 days. For this system, a degradation sequence of the carminic acid structure present in the effluent by the Tv fungus is suggested, in which it is seen that metabolites still containing aromatic structures are generated.

## INTRODUCTION

In recent years, there has been intense research regarding decolorization of dyeing effluents. It is known that the use of fungi, bacteria, and plants, through their enzymes, can help to reduce the amount of dye that is present in effluents[1,2,3,4,5,6,7] or toxic compounds within wastewater[8]. The application of biological processes that use fungi for effluent decolorization is one of the techniques currently in use. The most studied fungi are *Phanerochaete, Pleurotus, Bjerlandera, Trametes, Polyporus, Phelinus, Irpex, Funalia*, and *Thelephora*[9,10,11,12].

A wide variety of *Trametes* fungi species have been studied due to their capability to reduce the color of effluents containing synthetic dyes[13,14,15,16,17]. Particularly, the *T. versicolor* (Tv) species have been used in decolorization of synthetic dyes[13,14,15,16,18,19,20,21]. Work has been carried out with the polyanthraquinone dye (Poly R-478) in 200 mg $L^{-1}$ concentrations, resulting in color reductions ranging from 80 to 89%[19]. Likewise, with azo dyes and anthraquinone in 100–500 mg $L^{-1}$ concentrations, decolorization ranging from 91 to 99% has been achieved in batch reactors containing the DSM 11309 strain[16]. The aforementioned species have also been used on the decolorization of $B_{2GL}$ acid red (azo) and K-GR reactive blue (anthraquinone), in 100 mg $L^{-1}$ concentrations each, yielding decolorization percentages of up to 98% for the first dye and 99% for the second dye[20]. Another example is the color reduction in the *Amaranthus caudatus* colorant (50 mg $L^{-1}$) with the Tv ATCC20869 strain[21].

Despite extensive information regarding color reduction in effluents that contain synthetic dyes, little is known about the decolorization of effluents that contain natural dyes. The results of the studies on the ability of this ligninolytic fungus to decolor effluents led to evaluations of its potential regarding color reduction in a textile effluent with cochineal extract. Cochineal extract is a natural dye that contains carminic acid (20% ± 1). This acid is considered to be susceptible to the ligninolytic fungus because its structure is like that of a natural anthraquinone with a glycoside radical[22], which is similar to the composition of some reported synthetic dyes. Cochineal is commonly used worldwide by craft makers and small businesses to dye clothes, which generates colored effluents, and so we present an alternative for the treatment of such effluents. It is known that the use of fungi, bacteria, and plant enzymes can aid in the reduction of dye content in effluents[1,2,3,4,5,6,7]. Due to its effectiveness and low cost, biological treatments of dye effluents that use fungi are widely used[9,10,11,12]. The aim of this study was to establish under which conditions color reduction in the effluent of the cotton dyeing process by means of cochineal extract would be achieved, and to clarify the degradation sequence for the carminic acid that is present in the extract using Tv CDBB-H-1051 fungus.

## MATERIALS AND METHODS

### Effluent Characterization

The effluent was obtained from the cotton dyeing stage that uses cochineal extract. At this stage, the cotton fabric has undergone the washing and premordanting processes. The dry cochineal was obtained at the “Centro Tlapanochestli Oaxaca”, in Mexico, where it is used to dye Mexican craftwork/traditional clothing. The dyeing effluent contains a 2500 mg $L^{-1}$ cochineal concentration, which is equivalent to 500 mg $L^{-1}$ of carminic acid. The physicochemical characterization of this wastewater was carried out using procedures from the Standard Methods[23]. The parameters that were analyzed were pH, electrical conductivity, phosphorus ($P\text{-}PO_4$), total chemical oxygen demand (COD) and soluble chemical oxygen demand (SCOD), total suspended solids (TSS), volatile suspended solids (VSS), fixed suspended solids (FSS, minerals), and color (absorbance).

## Microorganism

Tv CDBB-H-1051 ligninolytic fungus was used, and was provided by the Culture Collection of the Biotechnology and Bioengineering Department at CINVESTAV-IPN. Petri dishes containing 2% malt extract were inoculated with the fungus and they were incubated at 28°C ± 1 for 5 days.

## Effects of the Effluent on the Fungal Growth in a Solid Medium

It is advisable to carry out preliminary tests of effluent decolorization in a solid medium with the intended organism since it will indicate if a liquid medium may be used[1,24]. Microbiological agar-agar (30 g $L^{-1}$) was dissolved in distilled water. Agar was sterilized at 103.42 kPa for 15 min, and was allowed to cool and later mixed with the corresponding effluent concentration of 5, 15, 30, and 65% (v $v^{-1}$), while another mixture without effluent and supplemented with nonsterile water was prepared. The effluent was taken directly from the dyeing process, without any prior treatment, following Standard Methods protocol[23]. Mixtures were poured into Petri dishes in triplicate. Once solidified, the fungi previously grown in malt extract were added. One centimeter diameter slices of agar-fungi were cut using a corkscrew, and they were inoculated into the Petri dishes with and without effluent. Fungal growth was measured diametrically every day and growth rate was estimated. All tests and measurements were conducted in triplicate.

## Effluent Decolorization Treatments in a Liquid Medium

Five 1-cm diameter slices of ligninolytic fungus were cut and placed into flasks with YPG (yeast-peptone-glucose) medium, and then they were stirred for 7 days at 28°C ± 1 and 125 rpm. Later, the obtained fungus was filtered and homogenized with a blender at medium level for 5 sec. An experiment with eight treatments was conducted (Table 1). Modified Kirk's medium was prepared[25] according to the treatments shown in Table 1. Sterilized distilled water was added to the treatments without Kirk's medium. To each of these, 50% (v $v^{-1}$) of nonsterilized effluent was added. The treatments that contained fungus received 5% (w $v^{-1}$) of inoculum and those that contained glucose received 5 g $L^{-1}$ (Table 1). In addition, a control was devised to determine if degradation could be attributed to the fungus or to the microorganisms in the effluent.

**TABLE 1**
**Treatments and Control for the Decolorization of a Textile Effluent in a Liquid Medium with CDBB-H-1051 Tv Ligninolytic Fungus, where 0 = Without; X = With**

| Treatment | Effluent (50%) | Kirk Medium (50%) | Glucose (5 g $L^{-1}$) | Fungus (5%) |
|---|---|---|---|---|
| 1 | X | X | 0 | 0 |
| 2 | X | 0 | 0 | 0 |
| 3 | X | X | X | 0 |
| 4 | X | 0 | X | 0 |
| 5 | X | X | 0 | X |
| 6 | X | 0 | 0 | X |
| 7 | X | X | X | X |
| 8 | X | 0 | X | X |
| Control | X | X | X | 0 |

There was a control with Kirk's medium and glucose, but without fungus, which underwent the same analysis as the other treatments, but was analyzed only at the beginning and at the end of the experiment (0 and 11 days). Measurements of pH, absorbance ($\lambda$ = 494 nm), and fungus dry weight after 0, 1, 4, 6, 8, and 11 days were done. Decolorization kinetics calculations were performed on treatments that presented that greatest reduction of color. Furthermore, chromatographic analysis was done to the initial and final samples of treatment 8 in order to determine conversion of carminic acid. The metabolites of the treatment that showed the greatest transformation of carminic acid were analyzed in each of the samples taken at the different times.

## Measurements of Fungus Dry Weight, pH, and Decolorization Percentage

Fungus dry weight was establish by the difference of weights between filter paper and filter paper plus fungus; both were dried in an oven at 100°C ± 1 for 24 h. Also, volume of filtered media was considered. pH was measured on a potentiometer at different times. In order to measure decolorization, absorbance was read (A) in a spectrophotometer (Jenway brand, model 6405, single beam), with the wavelength that corresponds to carminic acid ($\lambda$ = 494 nm). Percentage of decolorization was established considering the effluent's initial absorbance and the solution's absorbance after the treatment[20].

## Degradation Analysis of the Cochineal Extract Contained in the Effluent

Degradation of the cochineal extract that is present in the effluent was analyzed using a liquid chromatograph, Agilent brand, series 1200 (HPLC, high-performance liquid chromatography) with a UV-Vis detector, a diode array, a quaternary pump, an in-flow degasser, and a 5- to 100-µL autosampler. The operation variables for the system were flow rate, 0.6 mL $min^{-1}$; wavelength, 495 nm; introduced sample volume, 10 µL; column, Agilent Eclipse AAA 3.0 × 150 mm, with particle size being 3.5 µm. The mobile-phase composition consisted of an aqueous solution of phosphoric acid 1 mM (pH 2.5) and several acetonitrile concentrations according to time (0–15 min). The carminic acid retention time was 10.2 min. A 3000 Array Milton Roy spectrophotometer, model LR 45 227, was used to determine beforehand the work interval during which the chromatographic analysis was to be carried out.

In order to determine degradation rate, Newton's mathematical model was used (Fig. 1): $y = Ce^{-Kd(t)}$, where $Kd$ is the degradation rate in $day^{-1}$, $t$ is the time measured in day, $y$ is the mAU quantity that the HPLC measures at different times, and $c$ is a constants in mAU units.

## Toxicity Tests

A toxicity test was done on the treatment with the greatest discoloration and degradation (Experiment 8), at 0 and 11 days. This analysis was performed with *Vibrio fischeri* (*Photobacterium phosphoreum*) by NMX-AA-112-SCF1-1995, by an accredited laboratory in the city of Cuernavaca, Morelos, Mexico. The acute toxicity test using the luminescent bacteria, *P. phosphoreum*, is the exposure under controlled conditions to a population of bacteria[26]. The bacteria emit light in normal conditions, but with the presence of a toxin in the sample, the emission of light produced by the body decreases in proportion to the concentration of contaminants in the sample the effective concentration that kills 50% of the population (EC50)[26]. The effect is calculated in percentage terms compared to abate the emission of light[26]. Subsequently, to calculate toxicity units (TU), one hundred is divided between the median effective concentrations (100/EC50)[26].

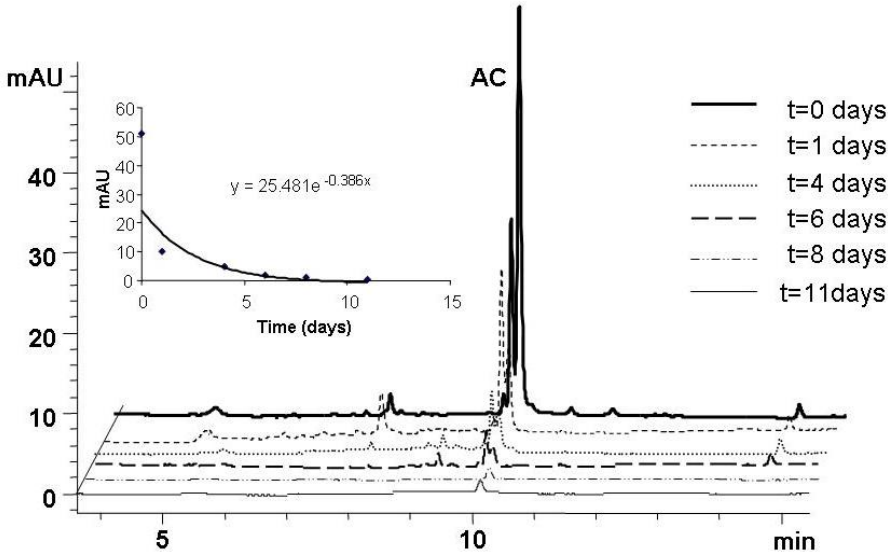

**FIGURE 1.** Kinetic degradation of the carminic acid in the cochineal extract for treatment 8, with Tv CDBB-H-1051. AC = carminic acid. Top left-hand side shows the degradation data (mAU), as a function of time following first-order model.

## RESULTS AND DISCUSSION

### Effects of the Effluent on the Fungus Development in a Solid Medium

Concentrations of carminic acid in Petri dishes with effluent were as follows: 0% (0 mg $L^{-1}$), 5% (25 mg $L^{-1}$), 15% (75 mg $L^{-1}$), 30% (150 mg $L^{-1}$), and 65% (325 mg $L^{-1}$). While analyzing fungus development in a solid medium, it was noticed that the Petri dishes with the lowest dye effluent concentrations showed greater radial fungus growth than those with the highest effluent concentrations (Fig. 2). Fungus growth did not show great differences within the 0 and 5% ($p > 0.05$, $\alpha = 0.05$) effluent concentrations, nor within the 15, 30, and 65% ($p > 0.05$, $\alpha = 0.05$) effluent concentrations. However, a significant difference was found between the group of 0 and 5% concentrations against the group of 15, 30, and 65% ($p < 0.05$, $\alpha = 0.05$) concentrations.

As fungus grew in the solid medium (agar effluent), color decreased, while thin filaments and a clear halo started to appear in the decolorization areas. The fungus thrived under these conditions and decolored the effluent. No growth of other microorganisms was detected in the Petri dishes even though the effluent had not been sterilized, suggesting a relation between fungus radial growth and effluent decolorization. These results were similar to those obtained by Eichlerová et al.[24], with the Tv CCBAS 612 strain, using synthetic dyes. Finally, the results achieved with fungus development in a solid medium led us to conclude that the effluent that contained cochineal extract could be decolored with the Tv fungus and proceeded to carry out the decolorization process in a liquid medium.

### Effluent Decolorization in a Liquid Medium

Treatments that contained fungus presented a higher color removal in comparison to those not inoculated. Treatment number 5 (Kirk’s medium without glucose), number 7 (Kirk’s medium with glucose), and number 8 (without medium, with glucose) presented high decolorization (81% ± 0.09; 88% ± 0.17; and 99% ± 0.04), and underwent decolorization kinetics calculations. From this point on, only those results will

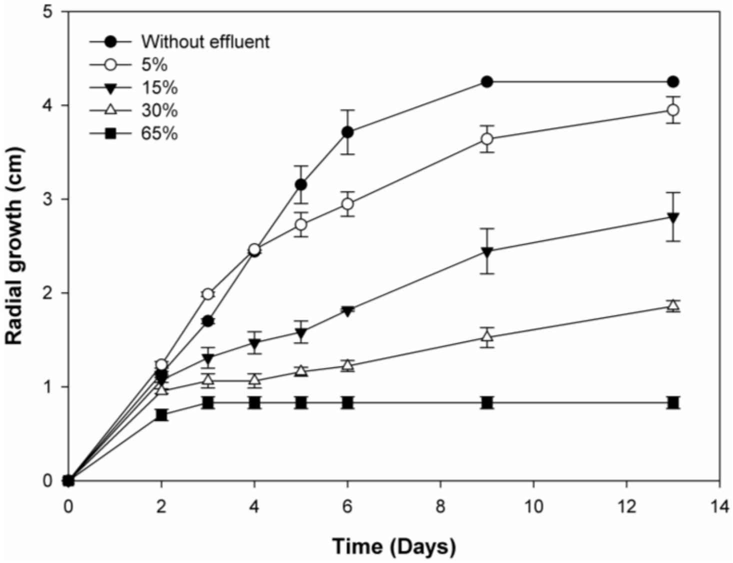

**FIGURE 2.** Radial growth of the Tv CDBB-H-1051 in Petri dishes with several concentrations of a textile effluent with cochineal extract. Bars represent standard deviation.

be discussed. Dry weight increased over time, with the exception of the control test, which remained the same throughout the experiment. The maximum dry weight measured was in treatment 5 (1.74 g $L^{-1}$ at day 1) and treatment 7 (2.38 g $L^{-1}$ at day 4). Treatment 8, without Kirk's medium, but with glucose, peaked on day 11 with a dry weight of 7.51 g $L^{-1}$. There was greater development of the fungus in treatment 8, indicating that the nutrient that probably stimulated growth was glucose. This is equivalent to what Ramsay et al.[21] found out regarding Tv fungal growth with glucose in the presence of the *Amaranthus caudatus* natural dye in the medium.

Decreases in pH were evident from the first day. There were not any pH adjustments during the experiment. For treatments 5 and 7, pH for both decreased from 4.9 to 4.0; then, it increased to 7.8 after 11 days. In treatment 8, pH almost reached optimal value for fungal growth at the end of the experiment (initial, 5.7; final, 4.1)[7,27]. This result is similar to that obtained by Ramsay et al.[21], where a change in pH in three different mediums was found when using the Tv fungus to decolor a natural dye. Treatment 8 yielded the highest effluent decolorization (99% ± 0.04), which suggests that decreased pH has some bearing on fungus development and decolorization efficiency. In treatments 5 and 7, decolorization was 81% ± 0.09 and 88% ± 0.17, respectively. The solution in these two treatments started to turn yellow, possibly due to Kirk´s medium's components influencing metabolism products. The final solution for treatment 8 (without Kirk's medium) was completely clear.

Additionally, a physicochemical characterization of the latter treatment was performed (Table 2). The greatest change was found in the percentages of solids; however, with a very small change for COD and the SCOD, which speaks of the existence of a high organic load. This is probably due to the presence of the ligninolytic fungus, in addition to the metabolites formed during the degradation stage.

Fig. 3 shows the chromatograms at the beginning (A, t = 0 days) and at the end (B–D, t = 11 days) of treatments 5, 7, and 8. It was noticed that treatment 8 showed a single peak at the end of the test, which is very close to that corresponding to carminic acid (Fig. 3D), while treatments 5 and 7 had about six and seven peaks, respectively (Fig. 3B and C), confirming that treatment 8 presented the best results regarding decolorization and degradation. The presence of glucose together with the absence of Kirk's medium allows for greater biodegradation of the effluent from the cochineal dyeing process. It is inferred that Kirk's medium contains compounds that affected degradation when compared to the treatment lacking Kirk's medium. A direct relationship between dry weight (fungal growth), pH, and decolorization of the

**TABLE 2**
**Characterization of the Effluent from the Cotton Dyeing Process with Cochineal Extract at the Beginning and at the End of Treatment 8 with Tv Fungus**

| Parameters | Beginning (t = 0 Days) | End (t = 11 Days) | Percentage of Reduction |
|---|---|---|---|
| TSS (mg $L^{-1}$) | 750 | 150 | 80.0 |
| VSS (mg $L^{-1}$) | 520 | 120 | 76.9 |
| FSS (mg $L^{-1}$) | 230 | 30 | 87.0 |
| Electrical conductivity (μS) | 435 ± 15 | 264 ± 2 | 60.7 |
| Color (absorbance, dilution 1:2) | 1.36 ± 0.02 | 0.01 ± 0.00 | 99.3 |
| pH | 5.7 | 4.1 | 28.1 |
| Fosfates (mg $L^{-1}$) (P-$PO_4$) | 12.80 | 8.40 | 34.4 |
| COD (mg $L^{-1}$) | 23,350 ± 397 | 20,250 ± 87 | 13.3 |
| SCOD (mg $L^{-1}$) | 21,350 ± 737 | 19,783 ± 29 | 7.3 |

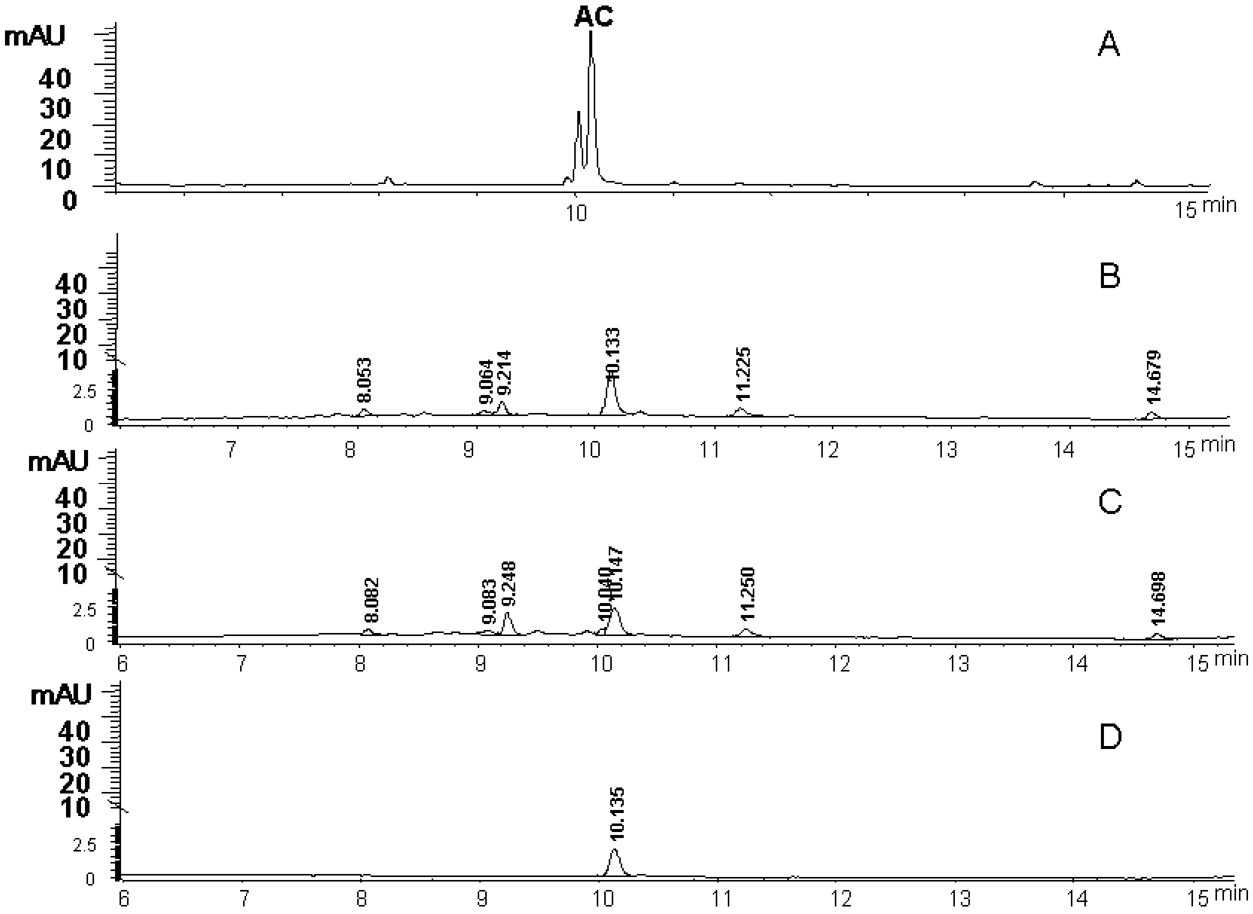

**FIGURE 3.** Chromatograms for treatments. (A) Initial, and after an 11-day period for treatments (B) 5, (C) 7, and (D) 8. AC = carminic acid.

cochineal effluent in the presence of glucose was observed, and graphed (Fig. 4), for treatment 8. No effluent decolorization was found in the control test, which contained Kirk's medium with glucose, but lacked fungus, proving the ability of the Tv fungus to reduce color in the effluent from the dyeing process. The results achieved in this study on removing color from an effluent of the cochineal dyeing process are comparable to those obtained by other authors regarding synthetic dyes[12,16], suggesting that this process may be applied to larger scales.

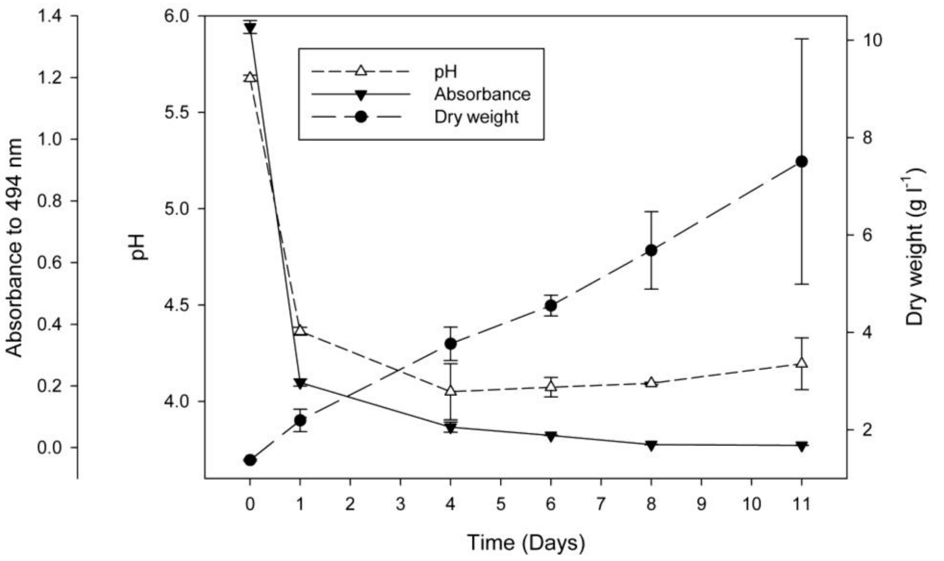

**FIGURE 4.** Relation between pH, dry weight, and absorbance at 494 nm for treatment 8 in the decolorization of an effluent with cochineal extract using Tv CDBB-H-1051. Bars represent standard deviation.

One of the main components of the cochineal extract that is present in the effluent is carminic acid. This acid is a natural anthraquinone that is attached to a glycosyl radical and has a great coloring power. It was deduced that the structure of anthraquinone from carminic acid was degraded by the ligninolytic fungus since there was no decolorization in the control without fungus and the chromatograms of the fungal treatments show a considerable decrease in the peak that corresponds to carminic acid ($t_R$ = 10.2 min, AC in Fig. 3a).

Fig. 1 shows the gradual disappearance of some peaks that were present in the effluent during the kinetics of treatment 8. In the initial chromatogram, the absorbance peak, which corresponds to carminic acid (AC, $t_R$ = 10.2 min), decreases as time passes by. The calculated degradation rate was $k_d$ = 0.39 day$^{-1}$. At other times, peaks that are very close to AC formed, suggesting the compound must also contain aromatic groups since conjugated aromatic compounds were seen under UV rays. This was confirmed with a liquid chromatography analysis, which found aromatic organic compounds, basically alcohols, ketones, esters, and organic acids. Therefore, a degradation sequence of AC with the Tv fungus is suggested (Fig. 5, compound number 1). 1-Carboxy 2-methyl 4,6,7,10-tetrahydroxy anthracene-3-8-dione; 3-4-dihydroxy phenol; 2-hydroxy 6-methyl benzoic acid; 1-methyl 3,5,8-trihydroxy 2-naphthalic acid; 1,2,3,4,5,6-penthahydroxy naphthalene; and hydroquinone can be detected by HPLC with diode array and correspond to the peaks that were noticed in the final chromatograms that were obtained for the treatments (t = 11 days) (Figs. 3 and 5). In the case of treatment 8, only one peak was detected in the chromatogram at the end of the experiment (t = 11 days) (Fig. 1). This suggests that the aromatic rings are partially degraded. This has been noticed before by Cajthaml et al.[28] for the degradation of anthraquinone-like compounds with ligninolytic fungus.

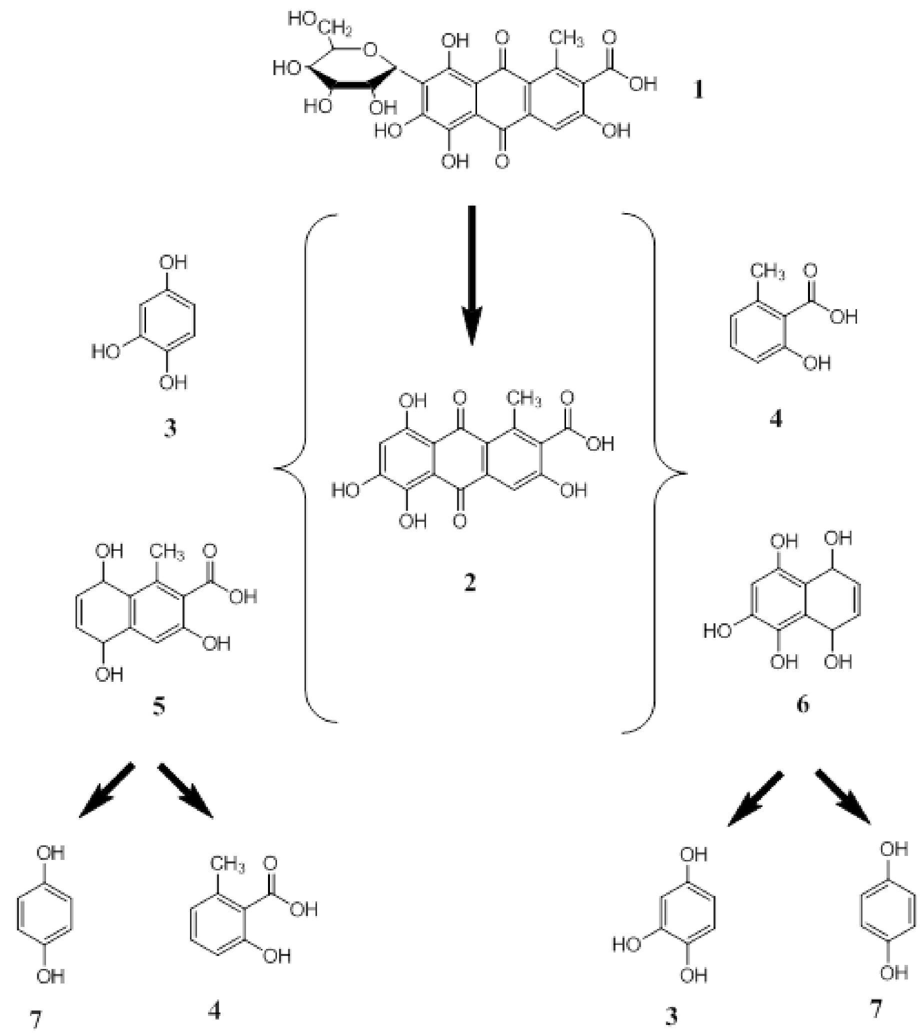

**FIGURE 5.** Main metabolites proposed for the degradation of the carminic acid structure, which is present in the cochineal extract effluent, using the Tv fungus (based on Cajthaml's proposal et al.[28]). 1 = Carminic acid; 2 = 1-carboxy 2-methyl 4,6,7,10-tetrahydroxy anthracene-3-8-dione; 3 = 3-4-dihydroxy phenol; 4 = 2-hydroxy 6-methyl benzoic acid; 5 = 1-methyl 3,5,8-trihydroxy 2-naphthalic acid; 6 = 1,2,3,4,5,6-penthahydroxy naphthalene; and 7= hydroquinone.

## Effluent Toxicity Testing

Toxicity analysis of the initial and the treated effluent helps to determine if the process produces toxic compounds. Toxicity analysis was done only on treatment 8, which had the highest rates of decolorization and degradation. The concentration of the sample that inhibits the production of light from the bacteria by 50%, $EC_{50}$[28], was 14% for the initial effluent and 2% by the end of treatment. Both values are considered highly toxic ($EC_{50}$ < 25%)[26]. Using toxicity units, 100/$EC_{50}$, TU[29], a value of TU > 4 is considered toxic[26]. For the initial effluent, TU = 7.33, and for the final effluent, TU = 47.73. Thereby, the toxicity between the initial and final effluent had an increase of 86%, yet treatment 8 also had the highest decolorization rate. The increase in toxicity is attributed to the change in pH that resulted at the end of the treatment (pH = 3.6). Gavril and Hodson[29] mention that the toxicity of a sample is due to its pH (determined by the chemical formula of the solute, which should be adjusted to between 6 and 8) and the inherent toxicity of the chemical components. In this case, the pH was not adjusted (3.6) in order to maintain the integrity of the samples. Hence, it is inferred that the high toxicity found was influenced by the final pH value, more so than the new compounds formed as a result of the degradation of the effluent[29]. The presence of phenolic metabolites in the final effluent treated with the Tv fungus (Fig. 5) are considered as the possible cause for the toxicity increase[30,31]. Therefore, a physicochemical treatment with the Fenton method is suggested to adjust the pH and reduce the toxicity, as the Fenton method is an efficient way of destroying complex molecular structures[32], such as those found during the decolorization treatment. This method also oxidizes compounds with relative ease, such as benzene and phenol[33,34]. Follow-up bench scale experiments to determine if this methodology can be done efficiently under the local craftwork industry conditions are planned.

## CONCLUSIONS

In a liquid medium, the Tv CDBB-H-1051 fungus eliminated the color from the effluent that contained the cochineal extract from the dyeing process. The fungus had a greater development and decolorization in the medium without Kirk's system and glucose (treatment 8). There is a direct relationship between the fungus dry weight, pH, and the system's decolorization, with higher efficiency at a lower pH. The carminic acid that is present in the effluent with cochineal extract was degraded by the Tv fungus, and contained aromatic structures with alcohol and acid functionalities (treatment 8). As the achieved results show, decolorization and degradation can be increased if pH is kept constant during the process at a favorable value for the fungal growth (pH 4.3), with pH adjustment of the final effluent needed before discharge to reduce its toxicity. This research established a protocol for degrading the carminic acid in the extract using Tv CDBB-H-1051 fungus. Also, it confirms that the fungus can be used for decoloring and degrading effluents that contain structures that are similar to the natural anthraquinones. This study establishes the conditions needed to reduce the color in the effluent from the cotton dyeing process with cochineal extract.

## ACKNOWLEDGMENTS

The authors would like to thank the Government of the State of Guanajuato and CONACYT through the "Mix Budget" for funding the project "The use of cochineal natural dye in the textile sector" (Code: GTO-04-C02-38). We would also like to thank SNI-CONACYT for their support in the project called "Alternatives for the treatment of effluents in the textile industry" (Code: CB3O070708) and partial support from Direccion de Apoyo a la Investigacion y al Posgrado, from the Universidad de Guanajuato with the project "Effect of inoculum on biological decoloration of cochineal dye effluent" (Code: 98/09). Gabriela Arroyo is also indebted to CONACYT-Mexico for doctoral scholarship Number 57295. We are especially grateful to Drs. Claudia Espinosa-Villegas and Pedro Lopez for their comments on the paper.
